# Late-Onset Depression and Dementia: A Systematic Review of the Temporal Relationships and Predictive Associations

**DOI:** 10.3390/medicina61050839

**Published:** 2025-05-01

**Authors:** Josheil Kaur Boparai, Megan Clemens, Khalid Jat

**Affiliations:** Faculty of Medicine, Memorial University of Newfoundland and Labrador, St. John’s, NL A1C 5S7, Canadakhalid.jat@nlhealthservices.ca (K.J.)

**Keywords:** dementia, Alzheimer’s disease, late-onset depression, systematic review

## Abstract

*Background*: Late-onset depression (LOD) has been increasingly recognized as a risk factor for dementia, yet the temporal and causal nature of this relationship remains unclear. *Objective*: The purpose of this review is to investigate the temporal association between LOD and dementia. *Methods*: A comprehensive search for studies examining the temporal relationship between LOD and dementia was conducted using MEDLINE via Ovid. The end date of the search was 9 September 2024. A total of 3450 studies were identified, of which 27 met the inclusion criteria. This review was conducted in accordance with the Preferred Reporting Items for Systematic reviews and Meta-Analyses (PRISMA) guidelines and an article quality assessment was completed. *Results*: The review demonstrated a significant temporal association between LOD and the risk of dementia, with the highest risk observed within the first decade following depression onset. LOD was consistently associated with an increased likelihood of developing dementia, particularly Alzheimer’s disease, compared to depression at earlier life stages. *Conclusions*: This systematic review highlights the significant association between LOD and dementia risk, emphasizing the need for early recognition and intervention. Future research should investigate the age at which LOD becomes a risk factor for dementia, the relationship between depression severity, family history of dementia, and dementia risk, as well as the efficacy of preventative treatments.

## 1. Introduction

Dementia is an age-related illness, acting as a major cause of disability and dependency for older people [1]. Worldwide, the elderly population is growing and thus, it is estimated that the number of people living with dementia will increase from 57.4 million in 2019 to 152.8 million by 2050 [2].

Currently, in Canada, over 700,000 people are living with dementia [3]. Altogether, it is clear that dementia is a significant and increasing factor that must be considered in the realm of scientific research and public health. Additionally, dementia poses a significant economic burden, wherein direct healthcare costs for Canadians with dementia are 3 times higher compared to those without dementia [4]. By 2031, Canada’s projected total healthcare costs and out-of-pocket caregiver costs will be upwards of $16 billion [4].

Given the projected increase in dementia diagnoses and the significant financial burden it poses, it is important to characterize the risk factors associated with cognitive decline to implement targeted interventions. Notably, recent research indicates that dementia in the prodromal phases and after disease onset cannot be cured or reversed, which underscores the importance of early identification of risk factors associated with dementia to help delay or prevent the onset of dementia [5]. While some risk factors, like advancing age, female sex, and genetic mutations in genes such as *PSEN1*, are not modifiable [6,7], it is important to identify those that can be altered. The 2020 Lancet Commission on dementia prevention, intervention, and care, identified 12 modifiable risk factors: Lower educational attainment, hearing impairment, smoking, hypertension, diabetes obesity, depression, physical inactivity, infrequent social contact, excessive alcohol consumption, head injury, and air pollution [8,9]. It is estimated that these 12 risk factors account for 40% of worldwide dementia cases [8]. Thus, it is of interest to identify further modifiable risk factors that can be screened for and addressed, if applicable.

Due to the common nature of depression, in the dementia population [10], the relationship between dementia and depression, and depression as well as depression as a potential modifiable risk factor, has long been a focus of research. Prevalence studies show that depression rates in mild, moderate, and severe dementia were 38%, 41%, and 37%, respectively [11]. Literature on this relationship has led to several hypotheses: (1) depression may serve as an independent risk factor for dementia; (2) depression could lower the threshold for dementia symptoms to emerge; (3) dementia or cognitive impairment might represent a depression characteristic; (4) depression could be an early indicator, or prodrome, of dementia; (5) depression may develop as a response to cognitive decline; and (6) depression and dementia might share common risk factors, accounting for their higher prevalence and frequent co-occurrence in certain populations [12]. The greatest evidence is for hypothesis 1; depression is an independent risk factor for dementia. However, this hypothesis can be further expanded to examine the impact of depression onset on dementia development. Late-onset depression (LOD) is defined as depression onset after age 60 or 65 years [13]. In the extant literature, LOD and late-life depression (LLD) are often used interchangeably, however, these terms are distinct. LLD refers to depression in those over 60 years, regardless of whether the first onset of depression was early in life or in later life [13]. This distinction is important as prior research emphasizes significant age-related differences in depression. Depression limited to adulthood or late life alone has been shown to not be associated with increased dementia risk after accounting for changes in symptoms over time [14]. However, persistent or recurrent depression across both adulthood and late life was linked to a higher risk of dementia, highlighting its potential role as a risk factor rather than a prodrome. These differences in depression onset have been further explored in neurocognitive and neuroimaging studies. For example, examination of neurocognitive profiles showed greater reductions in processing speed and executive function in older adults with LOD compared to early-onset depression (EOD) [15]. Similarly, MRI white matter hyperintensities were about 4.5 times more common among older adults with LOD compared to EOD [16]. Given these differences, our systematic review specifically focused on LOD and did not include studies examining LLD. This approach allowed for a more nuanced understanding of the unique association between first-onset depression in later life and dementia risk, accounting for its distinct clinical, cognitive, and neurobiological features.

The neurobiological mechanism mediating the link between depression and dementia is suggested to be the increase in hypothalamic-pituitary-adrenal (HPA) axis activity. This heightened activity leads to increased glucocorticoid production, which contributes to hippocampal atrophy and impairments in learning and episodic memory which eventually manifests as dementia [13]. Moreover, the impact of HPA axis dysregulation appears to be more pronounced in older adults with depression, who exhibit greater abnormalities in HPA axis function compared to younger individuals [17]. Chronic hypercortisolemia may impair neuronal functioning, particularly in the hippocampus, a key region implicated in both mood regulation and memory function [18]. Another contributory factor is the upregulation in pro-inflammatory processes (e.g., activation of microglia, changes in transforming growth factor beta 1 and downregulation of anti-inflammatory molecules which can also contribute to the subsequent development of depression and dementia [19]. These inflammatory changes may disrupt neurogenesis, synaptic plasticity, and neuronal survival, thereby contributing to both depressive symptoms and progressive cognitive decline [20].

In recent years, a growing number of studies have explored the link between LOD and dementia risk [21], but findings remain inconsistent. Despite the growing interest in the depression-dementia relationship, prior studies have several methodological limitations that must be addressed. Retrospective or cross-sectional designs are commonly used, making it inappropriate to draw temporal or causative conclusions [12]. Others fail to distinguish between EOD and LOD, despite evidence suggesting distinct neurobiological profiles [15,16]. Additionally, studies vary widely in how they define and measure depression and dementia, contributing to inconsistent findings. For example, they may rely on clinical diagnoses, prescription refills (for depression), and/or self-report measures, each of which captures a different perspective (and likely different prevalence) of these conditions. This introduces heterogeneity in both exposure and outcome definitions. A systematic review is essential to consolidate this mixed evidence and inform targeted public health policies and the development of preventive strategies to improve the quality of life and reduce healthcare costs associated with both LOD and dementia in elderly populations. By focusing specifically on LOD and excluding studies that do not distinguish the age of onset, the objective of this systematic review was to examine the extant literature for the purpose of determining the association between depression onset in late life and subsequent risk of dementia.

## 2. Methods

This systematic review was conducted in accordance with the Preferred Reporting Items for Systematic Reviews and Meta-Analyses (PRISMA) guidelines [22] and the PRISMA checklist was completed. This review was not registered and a review protocol was not prepared.

### 2.1. Search Strategy

MEDLINE via Ovid was searched on 1 March 2024 and again on 9 September 2024 (top-up search), due to its extensive coverage of biomedical and psychological literature, which is highly relevant to the focus of our research. While we acknowledge that additional databases, such as Embase and PsycINFO, could have potentially captured more studies, our search strategy was specifically designed to balance sensitivity and specificity within the scope of this review. By selecting MEDLINE as the main source, we aimed to focus on high-quality, relevant articles while ensuring the search remained manageable and aligned with the specific objectives of our investigation. Two concepts were searched for: ‘Mood disorders’ including depressive and bipolar disorders and ‘dementia’. The complete search strings are available in Appendix A.

### 2.2. Inclusion and Exclusion Criteria

Eligible studies focused on dementia as the primary outcome and its relationship to LOD. Dementia types considered were Alzheimer’s Disease (AD), vascular dementia, frontotemporal dementia, frontotemporal lobar degeneration, Lewy body disease, or Lewy body dementia. Depression was considered late-onset if the age of first diagnosis was 60 years old or greater. Leniency was allowed for this criteria; if the exact age was not included but most of the study participants met the age requirements, the study was included and results were interpreted with caution. LOD was specifically included for the purpose of understanding the temporality of this condition with dementia. Diagnosis of these conditions must have been established using valid instruments, such as self-report questionnaires or healthcare professional assessments. Studies lacking details on the diagnostic method for dementia or depression were excluded.

Only primary research articles were included; letters, correspondences, editorials, reviews, meta-analyses, commentaries, summaries, presentations, symposiums, focus groups, group discussions, case studies, case series, and observational studies were excluded.

Included studies reported associations between dementia and prior diagnosis of depression. Studies examining the treatment of dementia or mood disorders and its impact on other conditions were included. Studies focusing solely on prevalence, without addressing it as a primary outcome, were excluded.

Studies were limited to human participants and publications in English. No restrictions were placed on the year of publication, region, or patients’ ages, sex, ethnicity, or gender.

### 2.3. Study Selection Process

**Figure 1:**
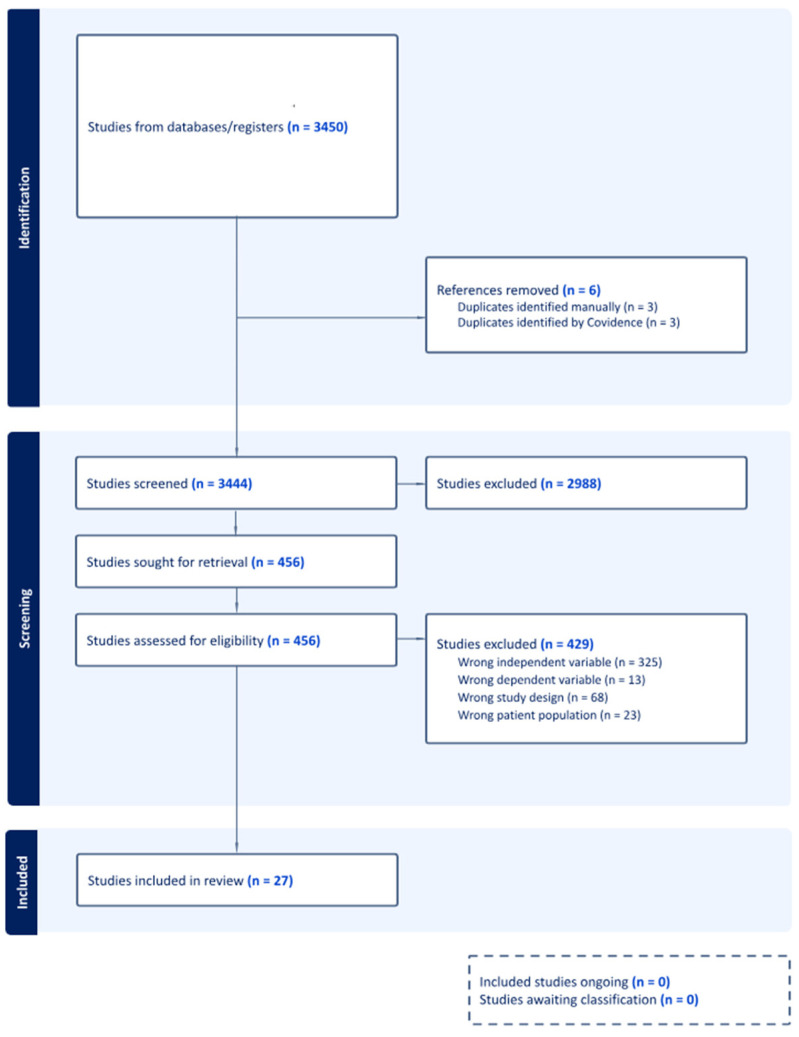
PRISMA flow diagram of studies screened and assessed for review.

Articles were uploaded into the Covidence software. Two authors (J.B. and M.C.) independently screened studies for inclusion. All studies first underwent Level 1 screening for title and abstract. Then, Level 2 screening involved screening the full text. Discrepancies were resolved by a third party (K.J.). A final number of 27 articles were selected for extraction (Figure 1).

### 2.4. Data Extraction and Synthesis

Data extraction was completed using Excel by one of two reviewers (J.B. and M.C.) using a data extraction template (Appendix B). Data synthesis was conducted by authors M.C. and J.B. using a six-phase thematic analysis methodology for analysis [23]. First, the authors re-read the extracted data and recorded initial ideas for thematic codes. Next, all items were systematically reviewed and manually coded. The coded and collated data was then sorted into potential themes, which were subsequently reviewed and refined. The fifth phase involved defining and naming the themes which were then used to complete phase 6 wherein, the extracted data was analyzed according to the corresponding themes. Any discrepancies about article inclusion at this point were resolved through discussion with K.J.

### 2.5. Quality Assessment

Quality assessment was conducted using the Joanna Briggs Institute critical appraisal tools, customized to each study design (e.g., cohort studies, case-control studies). Each article was assessed by one of two reviewers (J.B. and M.C.), answering up to 11 questions with “yes”, “no”, or “unclear”. Articles were included in the results regardless of quality. Detailed quality assessment results are provided in Appendix C.

## 3. Results

### 3.1. Temporal Association and Dementia Risk Magnitude

Five studies examined the temporal relationship between LOD and dementia, focusing on how the timing of depression onset relative to a dementia diagnosis influences dementia risk [24,25,26,27,28]. Table 1 summarizes the timing and magnitude of dementia risk associated with LOD across these studies. The association between LOD and dementia risk was found to be strongest within 3 months [26], 6 months [27], and 10 years of a depression diagnosis [24,25,28]. The risk of dementia dissipated over time, wherein after approximately 3 years, one study showed there was no significant difference in risk compared to non-depressed controls [26], whereas two studies found that the risk remained significant even after 20 years [25,27]. Interestingly, one study found that having a recent and past depressive episode was associated with higher odds of dementia (OR = 2.73, CI 1.08–6.87) [28].

### 3.2. Late-Onset Depression and Alzheimer’s Disease Risk

Eight studies examined the relationship between LOD and the risk of developing AD specifically [24,28,29,30,31,32,33,34]. See Table 2 for a brief summary of the findings of these eight studies. Three studies found that LOD increased AD risk [29,31,32]. Although another study also found an increased risk of developing AD in individuals with LOD (HR 1.71, 95% CI 0.62 to 4.74), this risk was less than half the risk observed in those with EOD (HR 3.70, 95% CI 1.43 to 9.56) [33].

Four studies examined the timing of depression onset relative to an AD diagnosis, with conflicting results [24,28,30]. Specifically, depression onset within 10 years of a dementia diagnosis significantly increased the likelihood of developing AD [24] whereas one study reported a higher risk with depression episodes more than 10 years prior [30]. According to [28], the combination of past and present depression, but not each individually, was significantly associated with AD risk. Interestingly, [34] found no significant correlation between the onset of AD and the onset of depression but observed an Increase in the incidence of depression in the 5 years prior to and after the onset of AD dementia.

### 3.3. Age of Depression Onset and Dementia Risk or Prevalence

Eighteen studies explored the relationship between the age of depression onset and dementia risk, of which some compared LOD with depression at other life stages, while others assessed specific risk factors, timing, or progression rates [14,25,26,29,31,32,33,35,36,37,38,39,40,41,42,43,44,45]. See Table 3 for a summary. Three studies found that LOD posed a much higher dementia risk than midlife [25,32,35] or recurrent depression [35]. These findings were further supported by [39] which reported an adjusted HR (aHR) of 1.46 (95% CI = 1.16–1.84) for all-cause dementia in individuals with LOD (age ≥ 50), while early-life depression was not significantly associated with dementia risk (adjusted HR = 1.10, 95% CI = 0.83–1.47). On the other hand, [33] found that EOD (age < 60) was associated with an increased risk of developing all-cause dementia (aHR = 3.37, 95% CI = 1.39–8.17), whereas this increased risk was less pronounced in individuals with LOD (aHR = 2.51, 95% CI = 1.08–5.85). Similarly, [44] showed that when compared to mentally healthy individuals, individuals with EOD had a higher risk of dementia development; but this risk was not evident when comparing mentally healthy controls with those who had LOD. In fact, [45] reported a non-significant yet increased odds of developing dementia in patients with LOD compared to those without. According to [43], midlife and LOD had similar effects on the odds of developing dementia with oRs of 2.72 and 2.05, respectively. Similarly, two studies reported an HR above 2 for dementia development in LOD compared to non-depressed controls [37]; and compared to those without LOD [41]. As shown in [36], there was an increased odds of all-cause dementia (OR = 2.16), and AD (OR = 1.57) among individuals with LOD, while highlighting that depression at any age elevated dementia risk. According to [26], incident depression at age 65 years or older doubled the incidence of dementia (IRR = 1.58, *p* < 0.01), but this risk was reduced (IRR = 1.11, *p* < 0.01) after adjusting for comorbidities. It was observed an unadjusted HR of 1.97 (95% CI = 1.36–2.85, *p* < 0.001) for dementia among individuals with LOD, though this association became non-significant after adjusting for baseline Geriatric Depression Scale scores [14]. Additionally, it was highlighted by [40] that family history and duration of the first depressive episode increased dementia risk in individuals with LOD. Interestingly [31] found that the risk of developing dementia increased with higher age cut-offs for LOD wherein onset at age 70 or older independently predicted all-cause dementia. However, one study found that regardless of depression onset (early, middle, or late), the hazard of dementia was more than 2 times greater compared to non-depressed controls, with the lowest hazard for LOD (2.31) [25].

The timing and progression of dementia in depressed individuals were investigated in three studies [29,38,42]. According to [38], authors reported a 19% dementia conversion rate among depressed elderly at two years, with a respective OR of 3.44 compared to non-depressed controls. Similarly, [29] observed a 24.7% dementia development rate among LOD cases over five years, significantly higher than in EOD (10%) or control groups (5.6%). Furthermore, [42] found that dementia prevalence was higher in the LOD group (47.5%) compared to the EOD (31.5%, *p* = 0.025).

### 3.4. Medication Use and Dementia Risk in Patients with Late-Onset Depression

Three studies investigated the relationship between specific medications and dementia risk in populations with LOD [46,47,48]. See Table 4 for a summary. According to [46] aspirin use was significantly associated with a reduced risk of dementia (aHR = 0.833; 95% CI 0.708–0.981, *p* = 0.029). Similarly, lipid-lowering agents (LLAs), particularly statins, were associated with a lower dementia risk [47]. Furthermore, [48] compared antidepressant use among individuals with LOD and found that antidepressant use did not significantly change the risk of dementia in those with LOD. These findings highlight the potential protective role of both aspirin and statins in mitigating dementia risk in individuals with LOD, with statins showing a particularly strong association.

### 3.5. Sex and Dementia Risk in Late-Onset Depression

Four studies highlighted sex differences in dementia risk associated with LOD [26,43,46,49]. See Table 5 for a summary. According to [26] a stronger temporal association was found between LOD and dementia in men compared to women. On the other hand, the remaining three studies found that female sex was a significant risk factor for dementia in individuals with LOD [43,46,49].

### 3.6. Age and Dementia Risk in LOD

Three studies emphasize the role of age in the relationship between LOD and dementia (see Table 6 for a summary) [26,46,49]. One study found that the temporal association between LOD and dementia was strongest in younger individuals (ages 65–74), followed by those aged 75–84, and least in those aged 85 and older [26]. Similarly, [46] identified age as a significant risk factor for dementia in patients with LOD. Another study further reinforced this finding, reporting that each additional year of age at the study baseline was associated with a 21% increased risk of developing dementia (HR = 1.21, 95% CI, 1.19–1.24) [49].

### 3.7. Late-Onset Depression Severity and Dementia Risk

Three studies examined the role of depression severity in dementia risk, with contrasting findings [27,29,35]. Table 7 provides a summary of these findings. According to [35] individuals with mild LOD, characterized by prescription renewals without hospital admission, had a higher HR (HR = 5.23) for developing all-cause dementia compared to those with severe LOD requiring hospital admission (HR = 3.14). On the other hand, ref. [27] found that severe LOD contributed to a higher risk of dementia compared to those with mild LOD. Specifically, severe depression showed a stronger association with vascular dementia compared to AD. In contrast, one study reported that the severity of depressive symptoms, whether minor or major in LOD, did not significantly influence dementia risk [29].

## 4. Discussion

This review identified two key findings: the increased risk of dementia associated with LOD compared to EOD, and the temporal relationship between LOD and dementia onset. LOD is consistently linked to a higher risk of dementia than EOD [29,32,35,38,39]. Specifically, depression onset at age 70 or greater independently predicts dementia risk [31], with LOD showing significantly greater odds of incident dementia compared to earlier-onset cases [36]. Importantly, the risk of dementia conversion is highest within the first six months following LOD onset [27], emphasizing the importance of early recognition and intervention. Clinicians should be vigilant in assessing LOD patients for dementia symptoms early and consistently, as timely intervention may help mitigate cognitive decline. Given the potential neuroprotective effects of certain medications, such as statins and aspirin, healthcare providers should explore personalized treatment plans that address both depression and dementia risk in this population. These findings align with prior literature highlighting age as the strongest predictor of dementia onset, even when depression status is not independently accounted for [6]. The established co-occurrence of depression and dementia likely stems from shared neuropathological pathways where decay contributes to both conditions [50]. This review underscores the temporal relationship between LOD and dementia, offering valuable insights into clinical management strategies. 

Findings on the association between LOD severity and dementia risk remain inconsistent. One study found that mild LOD had a higher risk of dementia than severe LOD [35]; another found severe LOD was correlated with vascular dementia (rather than AD) [27], and one found no association between LOD severity and dementia [29]. These results partly contradict prior literature on LLD (not restricted to LOD), which has established that patients with depression have double the risk of dementia [51]. Increased depression severity has also been consistently associated with higher dementia risk in earlier studies [52,53]. This discrepancy suggests that future studies should focus on disentangling the effects of depression severity, onset timing, and dementia subtype.

Medication use appears to modify the relationship between LOD and incident dementia. Aspirin and statins, for example, exhibit a protective effect against dementia onset in LOD patients [46,47]. Beyond the commonly prescribed acetylcholinesterase inhibitors [54], several other medications have been identified as protective. These include antihypertensive medications [55], disease-modifying antirheumatic drugs [56], as well as several anticonvulsants, antibiotics, and anticoagulants [57].These findings suggest that medications with neuroprotective or anti-inflammatory properties may influence dementia risk through shared neuropathological pathways. Further research should clarify the mechanisms underlying these associations and explore whether certain treatments could specifically mitigate dementia risk in LOD patients.

LOD is strongly associated with an increased risk of AD, particularly when depression occurs within 10 years of AD diagnosis [24,29]. However, some studies suggest that depression from 10 or more years ago also raises AD risk [30], while others indicate that only the combination of past and present depression significantly impacts AD development [28]. These findings highlight the need for a nuanced understanding of how the timing and chronicity of depression contribute to AD risk and progression.

Sex differences are evident in the relationship between LOD and dementia. Females with LOD generally exhibit higher overall dementia risk than males [43,46,49]. However, males show a stronger temporal association, with dementia occurring soon after depression diagnosis compared to females [26]. These findings suggest that sex-specific factors, such as hormonal or vascular differences, may influence the timing and magnitude of dementia risk in LOD patients.

The increasing age of depression onset is a clear risk factor for dementia. According to [49], each additional year of baseline age was associated with a 21% increase in dementia risk, supporting the idea that LOD reflects underlying neurodegeneration. While age is the strongest risk factor for dementia overall [6,58], the unique contribution of LOD timing merits further exploration to guide targeted prevention efforts.

### Strengths and Limitations

This review highlights the nuanced association between the timing of LOD diagnosis and dementia risk, rather than attributing risk solely to a general depression diagnosis. This review also includes large studies with robust methodology, enhancing the reliability of the findings. On a broader scale, the findings of this review highlight the importance of incorporating depression screenings as part of routine health assessment for older adults. Public health strategies should focus on raising awareness about the increased dementia risk associated with LOD. Programs that target the early identification of depression and its management could reduce the burden of dementia in aging populations.

A limitation of this study is insufficient evidence to recommend treatment regimes. This is in part due to the study design and the focus of the review being on associations rather than treatments. Study heterogeneity presents another challenge. For example, there was variability in how depression severity is defined, such as using prescription data versus validated scales, limiting comparability between studies. Finally, this review investigated correlation, not causation. Without casual evidence, the extent to which LOD contributes to dementia remains unclear. This limits recommendations for targeted interventions.

## 5. Conclusions

The association between LOD with dementia underscores the importance of prompt identification and management of LOD. While evidence regarding whether treated LOD reduces dementia risk remains inconclusive, addressing depressive episodes early may mitigate risk, especially given the temporal proximity between recent depression and dementia onset.

Educating patients with LOD about their increased risk of dementia is crucial. Interventions such as improving cognitive reserve through mentally stimulating activities, encouraging a healthy lifestyle, and considering preventative medications (e.g., statins or aspirin) could offer protective benefits. Furthermore, patients should be advised to monitor for early dementia symptoms to facilitate timely intervention.

Dementia profoundly impacts individuals, families, and, most broadly, healthcare systems. This review highlights a significant association between LOD and dementia risk, particularly regarding the timing of depression onset. Future research should investigate the critical age at which LOD becomes a risk factor for dementia; the relationship between depression severity, family history of dementia, and dementia risk; and the efficacy of preventative treatments.

## Figures and Tables

**Table 1:** Temporal Association and Dementia Risk Magnitude.

Study	Timing of Depression Onset Relative to Dementia	Key Findings
Heser et al., [26]	Within 3 months	Strongest association; no significant risk after ~3 years
Holmquist et al., [27]	Within 6 months; up to 20 years	Strongest early risk; significant risk persisted up to 20 years
Olazaran et al., [28]	Within 10 years; recent and past episodes	Strong association; recent + past depression → highest odds (OR = 2.73, CI 1.08–6.87)
Brommelhoff et al., [24]	Within 10 years	Strong association within 10 years
Elser et al., [25]	Within 10 years; up to 20 years	Persistent significant risk even after 20 years

***Note****:* OR: Odds Ratio; CI: Confidence Interval.

**Table 2:** Late-onset Depression and Alzheimer’s Disease Risk.

Study	Key Findings
Vilalta-Franch et al., [29]	LOD significantly increased AD risk
Barnes et al., [32]	LOD significantly increased AD risk
Heser et al., [31]	LOD significantly increased AD risk
Geerlings et al., [33]	LOD increased AD risk (HR = 1.71, CI 0.62–4.74), but less than EOD (HR = 3.70, CI 1.43–9.56)
Brommelhoff et al., [24]	Depression within 10 years of AD diagnosis increased risk
Speck et al., [30]	Higher AD risk with depression episodes >10 years prior
Olazaran et al., [28]	Past + present depression associated with AD; individually, not significant
Heun et al., [34]	No significant association between timing of depression and AD onset; depression ↑ within 5 years of AD onset

***Note***: LOD: Late-Onset Depression; AD: Alzheimer’s Disease.

**Table 3:** Age of Depression Onset and Dementia Risk of Prevalence.

Study	Comparison/Focus	Key Findings
Hickey et al., [35]	LOD vs. midlife/recurrent depression	LOD associated with much higher dementia risk
Barnes et al., [32]	LOD vs. midlife depression	LOD significantly increased dementia risk
Elser et al., [25]	Early/mid/late depression vs. controls	All increased dementia risk; LOD had lowest hazard (HR = 2.31)
Li et al., [39]	LOD vs. early depression	LOD (HR = 1.46); EOD not significant (HR = 1.10)
Geerlings et al., [33]	Early vs. late depression	EOD HR = 3.37; LOD HR = 2.51
Pálsson et al., [44]	Early vs. LOD vs. controls	EOD → higher dementia risk; no significant LOD risk
Zalsman et al., [45]	LOD vs. non-depressed	Non-significant but increased odds with LOD
Yu et al., [43]	Midlife vs. LOD	Similar odds: midlife OR = 2.72; LOD OR = 2.05
Kohler et al., [37]	LOD vs. controls	LOD HR > 2
Buntinx et al, [41]	LOD vs. no LOD	LOD associated with increased dementia risk
Yang et al., [36]	LOD, all-cause, Alzheimer’s dementia	LOD → OR = 2.16 (all-cause), OR = 1.57 (Alzheimer’s); all depression ↑ risk
Heser et al., [26]	Incident depression at 65+	IRR = 1.58; adjusted IRR = 1.11 (less after accounting for comorbidities)
Lee et al., [14]	LOD (65+)	Unadjusted HR = 1.97; not significant after adjusting for depression severity
Ohanna et al., [40]	Risk factors in LOD	Family history and duration of first depressive episode ↑ dementia risk
Heser et al., [31]	Age cutoff for LOD	Onset ≥70 independently predicted dementia
Tam et al., [38]	Conversion in elderly depressed (50+)	19% converted to dementia in 2 yrs; OR = 3.44 vs. controls
Vilalta-Franch et al., [29]	LOD vs. early vs. control	Dementia in 5 yrs: LOD (24.7%), EOD (10%), control (5.6%)
van Reekum et al., [42]	Dementia prevalence in LOD vs. EOD	LOD = 47.5%; early = 31.5% (*p* = 0.025)

Note: LOD: Late-Onset Depression; EOD: Early-Onset Depression

**Table 4:** Medication Use and Dementia Risk in Patients with LOD.

Study	Medication Examined	Key Findings
Yang et al., [46]	Aspirin	Reduced dementia risk (aHR = 0.833; 95% CI 0.708–0.981; *p* = 0.029)
Yang et al., [47]	Lipid-lowering agents (e.g., statins)	Statins significantly associated with lower dementia risk
Su et al., [48]	Antidepressants	No significant effect on dementia risk in individuals with LOD

**Table 5:** Sex and Dementia Risk in Patients with LOD.

Study	Key Findings
Heser et al., [26]	Stronger LOD–dementia association observed in males
Yang et al., [46]	Female sex identified as a significant dementia risk factor in LOD
Singh-Manoux et al., [49]	Females with LOD had higher dementia risk
Yu et al., [43]	Females with LOD exhibited elevated dementia risk compared to males

Note: LOD: Late-Onset Depression.

**Table 6:** Age-Related Differences in Dementia Risk Among Individuals with LOD.

Study	Key Findings
Heser et al., [26]	Strongest LOD–dementia association in ages 65–74, followed by 75–84, then 85+
Yang et al., [46]	Increasing age identified as a significant dementia risk factor in LOD
Singh-Manoux et al., [49]	Each additional year of age increased dementia risk by 21% (HR = 1.21; 95% CI: 1.19–1.24)

Note: LOD: Late-Onset Depression.

**Table 7:** Influence of Depression Severity on Dementia Risk in LOD.

Study	Key Findings
Hickey et al., [35]	Mild LOD (no hospitalization) had higher dementia risk (HR = 5.23) than severe LOD (HR = 3.14)
Holmquist et al., [27]	Severe LOD linked to greater dementia risk, especially for vascular dementia
Vilalta-Franch et al., [29]	Depression severity (minor vs. major) did not significantly impact dementia risk

Note: LOD: Late-Onset Depression.

## Data Availability

The original contributions presented in this study are included in the article. Further inquiries can be directed to the corresponding author.

## Abbreviations

The following abbreviations are used in this manuscript:

LOD	Late-Onset Depression
EOD	Early-Onset Depression
HPA	Hypothalamic-Pituitary Axis
AD	Alzheimer’s Disease

## Appendix A

### Search Strategy–Conducted in March 2024 and September 2024

Ovid MEDLINE(R) Epub Ahead of Print and In-Process, In-Data-Review & Other Non-Indexed Citations <1 March 2024>

Ovid MEDLINE(R) Epub Ahead of Print and In-Process, In-Data-Review & Other Non-Indexed Citations and Daily <9 September 2024>

## Appendix B

**Table A1:** Summary of the included studies.

Study (Author and Year of Publication)	Country of Study	Study Type	Sample Size	Age of Onset for LOD	Key Findings
Elser et al., [25]	Denmark	Retrospective Cohort	Depression: 246, 499 Without Depression: 1,190,302	≥60 years	Compared to controls without depression, individuals with LOD within 1–10 years, 10–20 years and 20–39 years had higher risk of dementia Overall during the follow-up period (1–39 years), the risk of dementia was higher among individuals with LOD compared to controls (OR of 20.1 vs. 17.3, *p* < 0.001) Compared to individuals with EOD (18–44 years) or middle-onset (45–59 years), patients with LOD had the highest dementia risks regardless of time elapsed between index date and depression diagnosis (1–10 years; >10–20 years; 20–30 years; 1–39 years). The hazard of dementia among those with LOD was low compared to those with early or middle-onset depression at 1–10 years, >10–20 years and 20–39 years since index date
Hickey et al., [35]	Denmark	Prospective Cohort	Total: 25,651 Depression: 8086	≥60 years	Compared to controls without depression, Danish nurses with LOD had a higher risk of dementia (HR: 5.85, CI: 5.17–6.64). This HR was higher than nurses with midlife only and recurrent (mid and late life depression)
Yang et al., [36]	Sweden	Cohort	Total: 41,727	>65 years	LLD depression was associated with increased odds of developing all-cause dementia (OR: 2.16), AD (OR: 1.57) and VaD (OR: 3.11). All other depression onsets were similarly associated with dementia.
Lee et al., [14]	Hong Kong	Prospective Cohort	Total: 16,608	≥65 years	Compared to controls without depression, individuals with LOD had a non-significant but lower hazard of incident dementia (HR: 0.91, *p* = 0.69). Only those with persistent depression (adulthood and LOD) showed a slightly increased hazard of dementia (HR: 1.13, *p* = 0.001)
Yu et al., [44]	Republic of Korea	Retrospective Cohort	Depression: 1824 Without Depression: 374,852	≥65 years	Compared to controls without depression, depression onset ≤44 years was not significantly associated with an increased odds of dementia. Compared to controls without depression, depression onset at 45–64 years and ≥65 years depression was associated with a significantly higher risk of developing dementia (OR 2.72, *p* = 0.003; OR 2.05, *p* = 0.001). The highest dementia risk was among Individuals with LOD.
Heser et al., [26]	Germany	Prospective Cohort	Total: 97,110	≥65 years	Incident depression diagnosis at age 65 or older increased subsequent risk of dementia (IRR = 1.11, *p* < 0.01). When stratified according to age at diagnosis (65–74, 75–84, and 85+), the risk of dementia was highest for the 65–74 year old age group (IRR = 2, *p* < 0.01). In older adults, the risk of dementia was significantly higher among those with depression compared to non-depressed individuals. Ages 65–74: Depression was associated with a 3.84-fold increased dementia risk, though this difference was not significant after 9 quarters. Ages 75–84: Dementia risk was 2.3 times higher in depressed individuals, with no significant difference after 8 quarters. Ages 85 and above: Depression was linked to a 1.34-fold increased dementia risk, with no significant difference after 3 quarters.
Yang et al., [47]	Taiwan	Retrospective Cohort	Depression: 6028 Without Depression: 40,411	≥65 years	Aspirin use among patients with LOD was associated with a lower risk of dementia (HR =0.734, 95% CI 0.641–0.841, *p* < 0.001)
Holmquist et al., [27]	Sweden	Retrospective Cohort	Cohort 1: Cases and Controls–Total = 238,772 Cohort 2: Siblings–Total = 50,644	≥50 years	Matched cohort: Odds of dementia were highest within 6 months of a depression diagnosis (aOR 15.20, 95% CI 11.85–19.50; *p* < 0.001), which decreased over time but persisted over 20 years of follow-up (aOR 1.58, 95% CI 1.27–1.98; *p* < 0.001) Sibling cohort: Odds of dementia were highest within 6 months of a depression diagnosis (aOR 20.85, 95% CI 9.63–45.12; *p* < 0.001), which decreased over time but persisted over 20 years of follow-up (aOR 2.33, 95% CI 1.32–4.11; *p* < 0.001)
Singh-Manoux et al., [50]	England	Prospective Cohort	Total: 10,308	Mean age: 70 years	LLD but not midlife (mean age 50 year) was associated with a higher risk of dementia
Yang et al., [48]	Taiwan	Retrospective Cohort	Total: 45,973	≥65 years	Among patients with LOD, compared to non-statin users, patients using statins had a lower risk of dementia (aHR = 0.674, 95% CI 0.547–0.832, *p* < 0.001). Among patients with LOD, patients taking a non-statin lipid lowering agent did not have a significant reduction in the hazards risk of dementia compared to those who did not use LLAs (HR 0.826, *p* = 0.117, 95% CI 0.65–1.049; HR 0.724, *p* = 0.349, 95% CI 0.369–1.422).
Kohler et al., [38]	The Netherlands	Retrospective Cohort	Total: 35,791	≥50 years	Unadjusted and adjusted hazard ratio showed that LOD was associated with a higher risk of dementia: (adjusted HR = 2.03, 95% CI = 1.56–2.64) compared to those without depression.
Tam and Lam, [39]	China	Case-Control	Depression: 81 Without Depression: 468	≥50 years	Compared to those without depression, LOD patients had an increased risk of progression to dementia at 2 years follow-up
Heser et al., [31]	Germany	Prospective Cohort	Total: 2663	≥60 years	LOD was associated with an increased risk of all-cause dementia (HR: 1.39, CI: 0.83–2.34), AD (HR: 1.53, CI: 0.75–3.12) and dementia of other etiology (HR: 1.24, CI: 0.54–2.80), albeit all were non-significant VLOD at ≥65 years was associated with an increased risk of all-cause dementia (HR: 1.65, *p* < 0.10). When the age cut-offs for VLOD were altered to 70 or 75 years, the risk associations increased Adjusted Models: VLOD increased the risk for all-cause dementia (HR: 1.51, CI: 0.86–2.65), Alzheimer’s Disease (HR: 1.31, CI: 0.58–2.95), and dementia of other etiology (HR: 2.05, CI: 0.89–1.97)
Vilalta-Franch et al., [29]	Spain	Retrospective Cohort	Total: 451	≥65 years	Compared to those with EOD, a greater proportion of LOD patients developed dementia (χ² = 2.847; df = 1; *p* = 0.092). Compared to those with EOD, a greater proportion of LOD patients developed Alzheimer’s disease (χ² = 8.475; df = 2; *p* = 0.014). Compared to those with no depression, Late-Onset Minor Depression or Dysthymia and Late-Onset Major Depression were associated with a greater risk of dementia development, which was further increased if patients had DEDS. LOD (Minor Depression or Dysthymia) without DEDS: HR: 1.565, *p* = 0.476 LOD (Minor Depression or Dysthymia) with DEDS: HR: 4.208, *p* = 0.001 LOD (Major Depression) without DEDS: HR: 1.653, *p* = 0.354 LOD (Major Depression) with DEDS: HR: 6.262, *p* = 0.002)
Olazaran et al., [28]	Spain	Retrospective Cohort	Never Depression: 1471 Past Depression: 85 Present Depression: 185 Present and Past Depression: 66	Present Depression = Within the last 10 years	prD (Present depression) and prpD (present and past depression) was associated with an increased risk of all dementia (OR = 1.84 (95% CI: 1.01–3.35), *p* < 0.05; OR = 2.73 (95% CI: 1.08–6.87), *p* < 0.05.). Past depression did not have a statistically significant association. Dementia due to AD showed non-significant associations with past depression and present depression. However, past and present depression showed a statistically significant association with dementia due to AD.
Barnes et al., [32]	USA	Cohort	Total: 13535	Defined late-life based on diagnoses between 1990–2000. Baseline age of participants was 40–55 years old in 1964–1973	Hazard of dementia was significantly higher among those with depressive symptoms in midlife only (HR 1.19 [95% CI, 1.07–1.32]), late-life only (HR 1.72 [1.54–1.92]), and those with both (1.77 [1.52–2.06]). Individuals with LLD symptoms had an increased risk of AD (HR, 2.06 [95% CI, 1.67–2.55]), and Vascular Dementia (HR 1.47 [1.01–2.14]). Individuals with midlife and LLDsymptoms had an increased risk of AD (HR, 1.99 [95% CI, 1.47–2.69]) and VaD (3.51 [2.44–5.05]).
Li et al., [40]	USA	Prospective Cohort	Total = 3410	≥50 years	LLD was associated with increased risk of all-cause dementia (aHR = 1.46, 95% CI = 1.16–1.84), but early life depression was not (aHR = 1.10, 95% CI 0.83–1.47)
Ohanna et al., [41]	Israel	Retrospective Case-Control	Without LOD: 51 LOD: 51	≥50 years	There was no significant association between LOD and dementia risk. However, a family history of dementia in patients with LOD (χ21 = 53, *p* = 0.022) and the duration of the first LOD episode (χ2101 = 1.8, *p* = 0.048) were associated with dementia development.
Brommelhoff et al., [24]	Sweden	Case-Control	Case Control: Controls = 12133; Cases = 547. Co-Twin Control = 146 Twin Pairs	Recent Onset: within 10 years of dementia onset Early onset: > 10 years prior to dementia onset	Case Control Analysis: Individuals with a recent onset of depression were 3.87 times more likely than those with no depression to have dementia (CI = 2.10, 7.14), and 2.62 times more likely to have AD (CI = 1.12, 6.17) Early onset of depression was not associated with an increased risk of dementia (OR = 0.90, CI = 0.44, 1.85) or AD (OR = 0.66, CI = 0.24, 1.81).
van Reekum et al., [43]	Canada	Cross-Sectional	N = 245; (N = 229 for admission MMSE N = 125 for discharge MMSE; N = 191 for admission MDRS)	≥60 years	A significantly greater proportion of patients with LOD (47.5%) developed dementia compared to those with EOD (31.5%) (*p* = 0.025)
Buntinx et al., [42]	Netherlands	Retrospective Cohort	LOD = 489 Without LOD = 18614	≥50 years	Compared to those without LOD, patients with LOD had a higher odds (OR 2.38, 95% CI = 1.08–5.06) and hazard ratio (HR 2.55, 95% CI = 1.19–5.47; *p* = 0.03) of dementia
Speck et al., [30]	USA	Case-Control Study	Control: 300 Dementia: 294	Depression < 10 years before dementia and ≥10 years	Depression occurring ≥ 10 years before dementia symptom onset increased risk of AD regardless of whether we looked at depression from any source (OR = 1.7, CI = 1.0–2.9) or restricted it to depression not related to loss/grief (OR = 2.4; CI = 1.2–4.5). However, depression occurring less than 10 years before dementia symptom onset did not increase risk of AD regardless of whether we looked at depression from any source (OR = 1.1, CI = 0.5–2.3) or restricted it to depression not related to loss/grief (OR = 1.0; CI = 0.3–2.8).
Geerlings et al., [33]	Netherlands	Population-based cohort study	Total = 486	≥60 years	Having a history of depression increased the risk of AD (HR 2.46; 95% CI 1.15 to 5.26). When history of depression was stratified by early and late onset, AD patients with EOD had increased risk of developing AD (HR 3.70; 95%CI 1.43 to 9.56) whereas this increased risk was less pronounced in subjects with a LOD (HR 1.71; 95% CI 0.62 to 4.74). When using all-cause dementia as the outcome, the results did not change.
Heun et al., [34]	Germany	Cross-Sectional	Total = 147	Stratified onset from the time of dementia diagnosis: 46–50; 41–45, 36–40, 31–35, 26–30, 21–25, 16–20, 11–15, 6–10, 0–5 years	There was a correlation between the age at onset of the depressive disorder and the age at onset of dementia (Pearson’s coefficient R = 0.447, *p* = 0.001)
Pálsson et al., [45]	Sweden	Retrospective Cohort	Controls = 227 Depression = 62	≥65 years	The incidence of dementia between the ages of 85 and 88 in mentally healthy individuals and in depressed individuals did not differ significantly. Early-onset depression, particularly early-onset MDD, is associated with a higher risk of dementia compared to mentally healthy individuals, with statistically significant findings for early-onset MDD. LOD and dysthymia do not show statistically significant associations with dementia incidence.
Su et al., [49]	Taiwan	Retrospective Cohort	Total: 56,154	60–80 years and > 80 years	Comparing antidepressant user (both high and low) to non-user -No difference in risk of dementia With high antidepressant use, dementia more likely in: male patients (HR: 1.23, 95% CI: 1.01–1.50), those older than 80 years (HR: 1.44, 95% CI: 1.02–2.04), and those with major depression (HR: 1.56, 95% CI: 1.11–2.20)
Zalsman et al., [46]	Israel	Cohort	Total: 502	≥50 years	OR for dementia in patients with LOD (vs. those without)–1.94 (95% CI 0.98 to 3.84, *p* = 0.06)

Note: EOD: Early-Onset Depression; LOD: Late-Onset Depression; VLOD: Very Late-Onset Depression; LLD: Late-Life Depression; HR: Hazard Ratio; OR: Odds Ratio; CI: Confidence Interval; MDD: Major Depressive Disorder; AD: Alzheimer’s Disease; VaD: Vascular Dementia; LLA: Lipid Lowering Agent. DEDS: Depression Executive Dysfunction Syndrome. “Late-life depression” is used in the above table to remain consistent with the language used in the authors’ original work.

## Appendix C

### Quality Assessment Tables

**Table A2:** Quality Assessment for all Cohort Studies (n = 22). **Colour Coding: Yes No Unclear N/A.**

Cohort Studies											
Study	Q1	Q2	Q3	Q4	Q5	Q6	Q7	Q8	Q9	Q10	Q11
Barnes et al., [32]	Yes	Yes	Yes	Yes	Yes	Yes	Yes	Yes	Yes	N/A	Yes
Heser et al., [31]	Yes	Yes	Yes	Yes	Yes	Yes	Yes	Yes	Yes	No	Yes
Kohler et al., [38]	Yes	Yes	Yes	Yes	Yes	Yes	Yes	Yes	Yes	No	Yes
Vilalta-Franch et al., [29]	Yes	Yes	Yes	Yes	Yes	Yes	Yes	Yes	Yes	No	Yes
Olazaran et al., [28]	Yes	Yes	Yes	Yes	Yes	Yes	Yes	Yes	Yes	No	Yes
Li et al., [40]	Yes	Yes	Yes	Yes	Yes	Yes	Yes	Yes	Yes	Yes	Yes
Buntinx et al., [42]	Yes	Yes	Yes	Yes	Yes	Yes	Yes	Yes	Yes	N/A	Yes
Elser et al., [25]	Yes	Yes	Yes	Yes	Yes	Yes	Yes	Yes	Yes	No	Yes
Heser et al., [26]	Yes	Yes	Yes	Yes	Unclear	Yes	Yes	Yes	Yes	No	Yes
Hickey et al., [35]	Yes	Yes	Unclear	Yes	Yes	Yes	Yes	Yes	Yes	Yes	Yes
Holmquist et al., [27]	Yes	Yes	Yes	Yes	Yes	Yes	Yes	Yes	Yes	Yes	Yes
Lee et al., [37]	Yes	Yes	Yes	Yes	Yes	Yes	Yes	Yes	Yes	Yes	Yes
Singh-Manoux et al., [50]	Yes	Yes	Yes	Yes	Yes	Unclear	Yes	Yes	Yes	Unclear	Yes
Yang et al., [48]	Yes	Yes	Yes	Yes	Yes	Yes	Yes	Yes	Yes	Unclear	Yes
Yang et al., [47]	Yes	Yes	Unclear	Yes	Yes	Yes	Yes	Yes	Unclear	Unclear	Yes
Yang et al., [36]	Yes	Yes	Yes	Yes	Yes	Yes	Yes	Yes	Unclear	Unclear	Yes
Yu et al., [44]	Yes	Yes	Yes	Yes	Yes	Yes	Yes	Yes	Unclear	Unclear	Yes
Geerlings et al., [33]	Yes	Yes	Yes	Yes	Yes	Yes	Yes	Yes	Yes	Unclear	Yes
Su et al., [49]	Yes	Yes	Yes	Yes	Yes	Yes	Yes	Yes	Yes	N/A	Yes
Pálsson et al., [45]	Yes	Yes	Yes	No	No	Yes	Yes	Yes	Yes	No	Yes
Zalsman et al., [46]	Yes	Yes	Yes	No	No	Yes	Yes	Yes	Yes	N/A	Yes

Q1: Were the two groups similar and recruited from the same population? Q2: Were the exposures measured similarly to assign people to both exposed and unexposed groups? Q3: Was the exposure measured in a valid and reliable way? Q4: Were confounding factors identified? Q5: Were strategies to deal with confounding factors stated? Q6: Were the groups/participants free of the outcome at the start of the study (or at the moment of exposure)? Q7: Were the outcomes measured in a valid and reliable way? Q8: Was the follow up time reported and sufficient to be long enough for outcomes to occur? Q9: Was follow up complete, and if not, were the reasons to loss to follow up described and explored? Q10: Were strategies to address incomplete follow up utilized? Q11: Was appropriate statistical analysis used?

**Table A3:** Quality Assessment for all Analytical Cross-Sectional Studies (n = 2). **Colour Coding: Yes.**

Analytical Cross-Sectional Studies								
Study	Q1	Q2	Q3	Q4	Q5	Q6	Q7	Q8
van Reekum et al., [43]	Yes	Yes	Yes	Yes	Yes	Yes	Yes	Yes
Heun et al., [34]	Yes	Yes	Yes	Yes	Yes	Yes	Yes	Yes

Q1: Were the criteria for inclusion in the sample clearly defined? Q2: Were the study subjects and the setting described in detail? Q3: Was the exposure measured in a valid and reliable way? Q4: Were objective, standard criteria used for measurement of the condition? Q5: Were confounding factors identified? Q6: Were strategies to deal with confounding factors stated? Q7: Were the outcomes measured in a valid and reliable way? Q8: Was appropriate statistical analysis used?

**Table A4:** Quality Assessment for all Case-Control Studies (n = 4). **Colour Coding: Yes No Unclear.**

Case-Control Studies										
Study	Q1	Q2	Q3	Q4	Q5	Q6	Q7	Q8	Q9	Q10
Tam and Lam, [39]	Yes	Yes	Yes	Yes	Yes	Yes	Yes	Yes	Yes	Yes
Ohanna et al., [41]	Yes	Yes	Yes	Yes	Yes	No	No	Yes	Yes	Yes
Brommelhoff et al., [24]	Yes	Unclear	Yes	Yes	Yes	Yes	Yes	Yes	Yes	Yes
Speck et al., [30]	Yes	Yes	Yes	Yes	Yes	Yes	Yes	Yes	Yes	Yes

Q1: Were the groups comparable other than the presence of disease in cases or the absence of disease in controls? Q2: Were cases and controls matched appropriately? Q3: Were the same criteria used for identification of cases and controls? Q4: Was exposure measured in a standard, valid and reliable way? Q5: Was exposure measured in the same way for cases and controls? Q6: Were confounding factors identified? Q7: Were strategies to deal with confounding factors stated? Q8: Were outcomes assessed in a standard, valid and reliable way for cases and controls? Q9: Was the exposure period of interest long enough to be meaningful? Q10: Was appropriate statistical analysis used?
